# Feasibility and Field Performance of a Simultaneous Syphilis and HIV Point-of-Care Test Based Screening Strategy in at Risk Populations in Edmonton, Canada

**DOI:** 10.1155/2013/819593

**Published:** 2013-12-19

**Authors:** Joshua Bergman, Jennifer Gratrix, Sabrina Plitt, Jayne Fenton, Chris Archibald, Tom Wong, Ameeta E. Singh

**Affiliations:** ^1^Alberta Health Services, Edmonton STI Clinic, 3B20 1111 Jasper Avenue, Edmonton, Alberta, Canada T5K 0L4; ^2^Public Health Agency of Canada, 130 Colonnade Road, A.L. 6501H, Ottawa, ON, Canada K1A 0K9; ^3^Provincial Laboratory for Public Health, 8440-112 Street, Edmonton, AB, Canada T6G 2J2

## Abstract

Few studies have evaluated the feasibility of delivering syphilis point-of-care (POC) testing in outreach (nonclinical) settings in resource rich countries. The objectives of the study were to evaluate the feasibility and diagnostic performance of performing both HIV and syphilis POC testing in outreach settings and to document new cases identified in the study population. 1,265 outreach testing visits were offered syphilis and HIV POC testing and 81.5% (*n* = 1,031) consented to testing. In our population, the SD Bioline 3.0 Syphilis Test had a sensitivity of 85.3% [CI (68.9–95.0)], specificity of 100.0% [CI (99.6–100.0)], positive predictive value (PPV) of 100.0% [CI (88.1–100.0)], and negative predictive value (NPV) of 99.5% [CI (98.9–99.8)]. Test characteristics for the INSTI HIV-1/HIV-2 Antibody Test had a 100.0% sensitivity [CI (39.8–100.00], 99.8 specificity [CI (99.3–100)], 66.7% PPV [CI (22.3–95.7)], and 100.0% NPV [CI (99.6–100.0)]. Four new cases of syphilis and four new HIV cases were diagnosed. In summary, at risk population seeking STI testing found POC tests to be acceptable, the POC tests performed well in outreach settings, and new cases of syphilis and HIV were identified and linked to treatment and care.

## 1. Introduction

In the late 1990s, Canada appeared to be on the verge of eliminating syphilis, as all but one province/territory had achieved rates of less than 0.5 per 100,000 population in 1997 [1]. In 2001, the reported rate of infectious syphilis started to increase rapidly, particularly among men, related to outbreaks occurring in large urban centres across Canada [2]. The majority of outbreaks across Canada have occurred among men who have sex with men (MSM) and individuals involved in sex trade, but other outbreaks have occurred among heterosexual persons not reporting risks associated with either of these populations [2, 3]. Between 1999 and 2008, the province of Alberta experienced the largest increase in the reported rate of infectious syphilis in Canada and resulted in the province declaring a syphilis outbreak in March 2007 [2, 4]. The reported rate of infectious syphilis in Edmonton, the second largest urban municipality in Alberta and with a population of over one million people for the census metropolitan area, was 8.1 per 100,000 in 2009, higher than the provincial rate of 7.4 per 100,000 [5]. The majority of cases in this ongoing outbreak were in heterosexual persons, but vulnerable populations such as MSM, people of aboriginal descent, sex workers, and people who inject drugs (IDU) have been disproportionately affected [5, 6]. In 2011, 53% of reported male cases in the Edmonton zone occurred among MSM [7]. In 2010, the number of cases of infectious syphilis reported in Alberta and in Edmonton declined and continued to decrease to a reported rate of 3.2 per 100,000 in the Edmonton area in 2011 [8].

The return of infectious syphilis in Alberta has the potential to impact HIV control as individuals with syphilis have an estimated two-to-five fold increased risk of acquiring HIV [9]. Additionally, HIV positive individuals may be more infectious when coinfected with syphilis [10]. The reported rate of new HIV diagnoses was 7.9 per 100,000 population in Edmonton in 2011, with the majority of male cases reported among MSM, a group also affected by the resurgence of infectious syphilis in this area [11].

Standard syphilis and HIV testing in Alberta involves the collection and transportation of a specimen to one of two central laboratories, where it can take up to 10 days to receive reports on newly identified infections that require confirmatory testing. A retrospective review done at the Alberta Health Services (AHS) Edmonton STI Clinic in 1999 showed that approximately 17% of persons did not return for HIV test results [12]. Point-of-care (POC) tests have been of particular benefit in remote or resource-limited settings that may lack the infrastructure for laboratory-based screening tests, in populations that are traditionally more difficult to reach and where immediate results can influence patient care [13–17]. Even in resource rich countries, the ability to conduct the test in nontraditional settings, the rapid availability of results (usually in <30 minutes) and the elimination of loss to follow-up for test results may make this testing approach preferable to centralized laboratory screening in some settings [18].

A POC test has the potential to allow timely counselling, referral, and management and, in the case of syphilis, immediate treatment [15–17, 19]. Although over a dozen commercially available syphilis POC tests are available in some regions of the world, there are currently no licensed syphilis POC tests in Canada [20, 21]. The SD Bioline 3.0 is a syphilis POC test that has shown comparable performance to standard tests when used in prenatal or high risk population in low income countries and at the point-of-care [22]. Presently, one HIV POC test is licensed for use at the point-of-care in Canada, the INSTI HIV-1/HIV-2 Antibody Test Kit. Although the kit has been evaluated in acute care settings in Alberta, where the rapid test is being performed in a laboratory setting, it has not been evaluated at the POC in nonclinical, community-based settings serving hard to reach population [23]. POC testing for syphilis has never been offered and evaluated in Alberta.

The primary objective of this project was to evaluate the feasibility of performing both HIV and syphilis POC testing in outreach settings in populations affected by the ongoing syphilis outbreak. HIV POC testing was included in the study as HIV status impacts the recommended treatment for infectious syphilis [24]. The secondary objective was to evaluate the diagnostic performance of rapid POC tests against standard laboratory tests. The tertiary objective was to document new cases of syphilis and HIV identified in the study population and their linkage to treatment and care. Our hypotheses were that POC testing would be acceptable to Edmonton's at risk population and that test results would be comparable to standard testing.

## 2. Materials and Methods

### 2.1. Study Location and Population

Between February 14, 2011 and August 28, 2012, the Edmonton STI Clinic outreach team (comprised of Registered Nurses (RN) and Outreach support workers) offered POC testing to individuals 18 years of age and older who consented to standard STI testing. Both syphilis and HIV POC testing were conducted on whole blood obtained from finger prick specimens. Individuals could participate more than once. Individuals who self reported they were HIV positive did not undergo HIV POC testing, but individuals self reporting a previous syphilis diagnosis were not excluded from syphilis POC testing. POC tests were offered to individuals at outreach locations including two correctional facilities, three inpatient addictions facilities (one site is a male only residential program), one health centre, several community-based organizations such as inner city drop-in centres, organizations serving sex trade workers, and four sites visited by MSM (e.g., bathhouse and gay bars). These sites were selected as they were existing outreach sites where STI testing was already offered on a routine basis and did not require additional logistical or human resource support; however, they also provided access to the key populations that have been represented in the Alberta outbreak to date, such as Aboriginal people, people who inject drugs, those involved in the sex trade, and MSM who have made up the majority of reported male cases since 2010 in Edmonton.

Based on previous testing conducted by the STI Outreach team, we estimated that the prevalence of reactive syphilis tests among our population would be 6.3%. At 6.3% reactivity and with 1,000 individuals enrolled, we estimated that we would be able to identify a POC test sensitivity of 87.5% (±8%).

### 2.2. Testing

POC test results were compared to standard laboratory screening tests performed at the Alberta Provincial Laboratory for Public Health (ProvLab) on serum samples collected simultaneously from participants.

#### 2.2.1. Syphilis

The POC syphilis test used was the SD Bioline Syphilis 3.0 (SD Bioline 3.0 Test, Standard Diagnostics, Inc., Korea), which is a solid phase immunochromatographic assay for the qualitative detection of antibodies of all isotypes (IgG, IgM, and IgA) against *Treponema pallidum* (TP), with an expected result within 5–20 minutes [25]. The standard laboratory test used for syphilis was a treponemal-specific enzyme immunoassay (EIA) (Architect Syphilis TP Microparticles, Abbott Laboratories, Illinois, USA), which was the initial screen [26]. Quantitative RPR titre was obtained on all samples tested reactive by the syphilis EIA (or Syphilis POC test) [26]. A line immunoassay (INNO-LIA Syphilis, Innogenetics NV, Ghent, Belgium) was used as the confirmatory test for samples submitted from individuals with no history of confirmed syphilis serology [26]. The EIA was used as the reference test for individuals who had a negative POC test (with no previous history of syphilis diagnosis and treatment) and for those with a positive POC test with a previous history of syphilis diagnosis and previously confirmed positive with the INNO-LIA. The INNO-LIA was used as the reference test for individuals with a positive POC test and no previous history of syphilis diagnosis and not previously confirmed positive with the INNO-LIA.

#### 2.2.2. HIV

The HIV POC test used was the INSTI HIV-1/HIV-2 Antibody test (INSTI HIV-1/HIV-2 Antibody Test, bioLytical Laboratories, Richmond, Canada). This is a manual, visually read, flow-through immunoassay for the qualitative detection of antibodies to HIV Type 1 and Type 2 in human EDTA-whole blood, fingerstick blood, serum, or EDTA-plasma in as little as 60 seconds [27]. For HIV, a third generation EIA (AxSym HIV 1/2 gO, Abbott Laboratories, Illinois, USA) was the initial standard laboratory screening test [26]. On June 14, 2011, ProvLab began using a fourth generation EIA (Architect HIV Ag/Ab Combo, Abbott Laboratories, Illinois, USA) as the initial screening test [26, 28]. Samples tested reactive by standard EIA screen or by HIV POC testing from individuals with no history of confirmed HIV serology were tested by a second EIA (Biorad Genetic Systems HIV-1/HIV-2 PLUS O EIA, BioRad Laboratories, California, USA) and HIV Western Blot (Genetic Systems HIV-1 Western Blot, BioRad Laboratories, California, USA) [26].

#### 2.2.3. Quality Control

In order to ensure the integrity of the testing materials, a control and proficiency testing program was established by the Laboratory Study Coordinator. Upon receipt of a new lot number of rapid kits, a lot release panel was performed and evaluated prior to the release of kits to storage. Kits were stored in the laboratory where temperatures were monitored on a daily basis. Additionally, the Laboratory Study Coordinator would run positive and negative controls on each new box of kits prior to distribution to the Outreach Team. The Provincial Laboratory also participated in a College of American Pathologists survey three times a year, thereby validating the proficiency of the supporting laboratory.

### 2.3. Patient Management

Algorithms for both syphilis and HIV POC testing were developed to guide testing, treatment, partner notification, and reporting. Syphilis treatment and counselling were based on the POC test result, previous syphilis testing and treatment, presence of signs and symptoms suggestive of syphilis, and the participant's sexual history. For those testing positive for syphilis by POC testing, the study nurse would contact staff at the STI Clinic to access the provincial STI database for a prior history of syphilis diagnosis and treatment in Alberta. All positive syphilis tests without a prior history of treatment were reviewed by phone with a STI consultant (AES) and a decision was made to provide immediate treatment or not. The POC syphilis test result was just one factor in the clinical decision algorithm for immediate treatment. Given the relative safety of treatment (benzathine penicillin G-long acting [Bicillin-LA]) and the potential harm posed by untreated syphilis, it is expected that the benefits of potential overtreatment outweigh any potential harm, especially in the context of hard-to-reach population who are more likely to be lost to follow-up. For those testing positive for HIV through POC, the result and case were reviewed by phone with a STI consultant (AES) to determine whether any immediate referral or management was required. Patients were informed that the syphilis or HIV POC test result was “preliminary” and the parallel standard testing being performed would confirm the result. All individuals with positive standard test results were contacted and actively followed up for necessary treatment and referral. For HIV positive results, this included referral to an HIV clinic.

### 2.4. Data Collection and Analyses

Standardized data collection forms were used by the nursing staff to collect and record demographics, clinical, and risk information on clients through a verbal history. All data was then entered into a password protected Microsoft Access database.

Feasibility was defined as the proportion of individuals consenting to POC divided by the total number of participants offered POC testing. To determine differences between participants consenting and those declining POC testing, age, gender, and testing location were compared using chi-square for categorical variables and Mann-Whitney *U* test for age. A two sided *P* value of <0.05 was considered statistically significant. A descriptive analysis of those individuals who had both syphilis and HIV POC testing was completed and due to the gender based differences in sex and drug behaviour, gender stratified analyses were also conducted to better understand our patient population. These analyses were performed using IBM SPSS Statistics 19 (IBM, Armonk, New York, USA). Diagnostic performances of POC test results were compared to standard testing results and test performance characteristics and 95% binomial confidence intervals were calculated (Stata version 11.0, StataCorp LP, College Station, Texas, USA). Cases were defined as having a new infection if the provincial databases for HIV and STI had no previous record of infection.

### 2.5. Ethics/Approvals

Ethics are obtained from the University of Alberta and Health Canada/Public Health Agency of Canada Research Ethics Boards. Special Access Approval was granted from Health Canada's Medical Devices Special Access Programme for the SD Bioline Syphilis 3.0 Test. Approval was obtained to provide POC treatment for syphilis based on a combination of the SD Bioline Syphilis 3.0 Test result as well as clinical information available at the time of testing (see Section 2.3).

## 3. Results

### 3.1. Feasibility of POC Testing

A total of 1,183 individuals at 1,265 outreach testing visits were offered syphilis and HIV POC testing and 81.5% (*n* = 1, 031) consented to testing. The majority of visits took place in locations where clients resided at the time of testing, correctional facilities (50.2%; *n* = 635) and inpatient addictions facilities (26.8%; *n* = 339). Ten percent (*n* = 126) of testing visits took place in locations serving MSM (Table 1).

There was no difference in gender or median age among visits where the participant consented to POC testing compared to those visits where POC testing was declined. Acceptance varied between testing sites with the highest acceptance among testing sites for MSM and the lowest acceptance at community-based organizations.

### 3.2. Characteristics of Study Population

In 1,031 visits, participants consented to either syphilis and/or HIV POC testing: in 1,024 visits, syphilis POC testing was performed (2 visits declined syphilis POC testing and 5 visits syphilis POC testing kits were not available) and in 1,012 visits, HIV POC testing was performed (11 participants declined HIV POC testing; 5 were previously positive for HIV and therefore did not meet inclusion criteria, and in 3 visits, HIV POC testing kits were not available). In 1,004 visits, both syphilis and HIV POC testing was performed on 951 individuals (Table 2). The majority of participants were male (73.3%), reported a history of substance use (non-IDU: 81.5% and IDU: 25.6%), shared of drug equipment (58.5%), and had previously been tested for HIV (82.6%). Nearly one-half of female participants (44.5%) reported a history of sex trade and nearly one-quarter (22.1%) of males reported sex with a sex trade worker. Nearly one-fifth (18.9%) of male participants reported the same sex partnering. Gender-stratified analysis indicates that in comparison to males, female participants were more likely to be younger (median 29 years versus 30 years *P* = 0.04), Gender-stratified analysis indicates that in comparison to males, female participants were more likely to be younger (median 29 years versus 30 years *P* = 0.04), be of Aboriginal descent (57.7% versus 39.0%, *P* < 0.001), report a history of IDU (35.4% versus 22.0%, *P* < 0.001), and to be previously syphilis seropositive (7.1% versus 1.9%, *P* < 0.001).

### 3.3. Diagnostic Performance of POC Tests

Five syphilis POC tests (0.49%) were false negatives when compared to the standard laboratory test; four were in individuals previously treated for syphilis and one was newly diagnosed, staged, and treated for late latent syphilis. There were no false positive syphilis POC test results.

Compared with standard serological testing, the SD Bioline 3.0 Syphilis Test had a sensitivity of 85.3% [CI (68.9–95.0)], specificity of 100.0% [CI (99.6–100.0)], positive predictive value (PPV) of 100.0% [CI (88.1–100.0)], and negative predictive value (NPV) of 99.5% [CI (98.9–99.8)] (Table 3).

Compared with standard serological testing, the INSTI HIV-1/HIV-2 Antibody Test had a 100.0% sensitivity [CI (39.8–100.00], 99.8 specificity [CI (99.3–100)], 66.7% PPV [CI (22.3–95.7)], and 100.0% NPV [CI (99.6–100)], when compared to standard serological testing (Table 3).

### 3.4. New Cases and Linkages to Care

Of 1,024 syphilis POC tests, 29 (2.8%) were positive, and of these, 25 (86.2%) were in individuals previously treated for syphilis with no evidence of a new syphilis infection (Table 3). The remaining four positive tests were in individuals with no previous history of syphilis diagnosis and treatment. Two of the individuals, one tested in a community-based setting and the other incarcerated, were treated presumptively at the time of testing and were subsequently staged and treated as early latent and late latent syphilis, respectively. The other two individuals, who were incarcerated and not considered at risk of being lost to follow-up nor at risk of transmitting syphilis before treatment, had their treatment delayed (5 and 19 days, resp.) to ensure adequate staging and treatment. These cases were subsequently staged as early latent and late latent syphilis and were treated while incarcerated.

Of 1,012 HIV POC tests, 6 (0.6%) were reactive; four were newly diagnosed HIV cases as confirmed by Western Blot and two were false positive test results, confirmed HIV negative by the BioRad EIA and Western Blot (Table 3). All four individuals were informed of their positive standard HIV test result within two weeks and were referred to an infectious disease specialist for HIV care.

## 4. Discussion

Our study is the first pilot of dual syphilis and HIV POC testing in outreach settings among at risk populations in North America.

### 4.1. Feasibility of POC Testing

Results from our study suggest that POC tests for syphilis and HIV were well accepted among at risk population in outreach settings in Edmonton. A significant proportion of individuals who consented to syphilis and HIV POC testing were incarcerated and reported substance use and sex work. The proportion of risk behaviours among our participants was similar to that individuals diagnosed with infectious syphilis in Edmonton during the outbreak [6].

The settings that had the highest acceptance of testing were those accessed by MSM such as a bathhouse, with 91.3% consenting to POC testing. The high acceptance of POC testing at MSM sites is supported by American research which showed significantly higher acceptance of rapid HIV testing than standard testing when offered in a bathhouse setting [29]. Almost half of the participants in this study were incarcerated and this group had a high acceptability of POC testing, as 80.0% consented to POC syphilis testing. This compares well to a US study, which showed 88% of inmates consenting to rapid HIV testing in a Rhode Island Department of Corrections jail [30]. The second most common setting where POCT was offered in our study was inpatient addiction treatment centres, where 85.8% of individuals offered POCT consented. This is higher than that of two US studies, which found 62% and 74% of individuals, respectively, consenting to rapid HIV testing in two separate inpatient substance abuse treatment programs [31, 32]. The higher rate of acceptance in this study could be related to POC testing only being offered to individuals who had already requested standard STI testing in outreach locations while the two US studies offered rapid testing to all individuals within their respective programs.

Although the reasons for declining POC tests were not examined, we found that our lowest acceptance rate was in community-based organizations. Numerous studies in North America have cited reasons for declining rapid HIV testing, particularly among high risk population in outreach settings, including the extra time taken to conduct the POC test, not being prepared to receive a HIV result the same day as the test, recent testing, the perception that they were not at risk for HIV infection, and the venue for testing [30–33]. Future research should evaluate the acceptability of syphilis POC testing, among at risk populations in outreach settings.

### 4.2. Diagnostic Performance of POC Tests

Multiple studies in the USA and Mexico have evaluated the technical performance of various rapid syphilis testing platforms [34–37]. The performance of the syphilis and HIV POC tests used in this study is similar to that reported in other studies [22, 23, 38]. A prospective multicentre clinic-based evaluation of the SD Bioline 3.0 Syphilis Test showed sensitivity ranging from 85.7 to 100% and specificity ranging from 98.1 to 99.4%, using whole blood in a clinic based setting, across four different sites [22]. A recent systematic review and meta-analysis of treponemal rapid POC tests for syphilis reported a sensitivity of 84.50% (78.81, 92.61) and specificity of 97.95% (92.54, 99.33) for the SD Bioline 3.0 Syphilis Test [38]. This compares to a lower sensitivity range of 85.3% (68.9–95.0) reported in this study. This might not be related to the test itself but rather to the environment or operator of the kit, for example, suboptimal sample volume being applied to the kit [19]. The lower sensitivity range reported in this study for the syphilis POC test would be a potential risk of missed opportunity for treatment. The field performance of the INSTI HIV-1/HIV-2 in this study was comparable to the report of a pilot study in Alberta, when the test was carried out in a controlled laboratory setting [23].

### 4.3. New Cases and Linkages to Care

We had hoped that syphilis POC testing would identify new infectious syphilis cases and allow POC treatment in affected populations in our region and thus assist with outbreak control. However the majority of individuals testing positive using the syphilis POC test in this study had been previously diagnosed and treated for syphilis (25 of 29 positive tests) and only four newly diagnosed cases of syphilis were identified (two cases of early latent and two cases of late latent syphilis). Half of the newly diagnosed cases received immediate treatment and in the other half, treatment was deferred but completed at a later date, as these participants were not at a risk of being lost to follow-up due to incarceration and not having an imminent release date. These data highlight the limited usefulness of treponemal POC tests in providing POC treatment to a previously syphilis seropositive population as the test is unable to distinguish between new and old infections. Unnecessary treatment may be provided unless quantitative nontreponemal testing is available when testing previously treated cases [39]. None of the 25 previously diagnosed syphilis cases identified in this study by POC testing were retreated because the testing RN was able to access the provincial STI database at the time of care to verify previous diagnosis and treatment. However in many field settings, the ability to verify previous diagnosis and treatment may not be possible. We postulate that POC testing in this outreach setting would have identified more cases in the earlier stages of the outbreak which peaked in 2006 at 10.9 per 100,000 but had declined to 3.4 per 100,000 and 3.2 per 100,000 in 2011 and 2012, respectively [8]. Dual treponemal and nontreponemal tests have the potential to address the issue of distinguishing previous infections from new infections. A recent study in China reported good test performance of a dual syphilis POC test when used in STI Clinic and outreach settings [40]. Future research is needed to evaluate the impact of using the dual syphilis POC test on uptake and intervention among high risk groups. The treponemal SD Bioline Syphilis 3.0 test was chosen for this study as no reliable dual syphilis POC test was available at the time the study was initiated.

The World Health Organization recommends assessing the existing quality of and access to syphilis testing and treatment for a population in determining the utility of introducing syphilis POC testing [41]. Standard syphilis testing is widely available in Alberta and is performed in accredited laboratories by skilled staff. However, the Alberta syphilis outbreak highlighted that those at highest risk do not necessarily access traditional testing settings, as evidenced by the increase in congenital syphilis cases, many of whom were born to street-involved women who had not accessed prenatal care which includes screening for syphilis [42]. The Outreach Team performing this study was formed in response to this observation and it was hoped that this population would be reached more readily and provided access to testing and ideally POC diagnosis and treatment of syphilis, thereby mitigating transmission.

In 2011, it was estimated that 25% of individuals living with HIV in Canada were unaware of their HIV status and not connected to necessary treatment and counselling services [43]. Nearly one-quarter of participants reported no previous HIV testing. This study identified four new HIV cases by HIV POC testing; all had reported previously testing negative for HIV, which highlights the importance of regular testing of high risk populations. All four newly diagnosed cases were confirmed positive by standard testing, informed of their standard test result, and successfully referred to a HIV clinic for ongoing care and treatment. Overall, one in five male study participants reported sexual contact with the same sex and three of the four newly identified HIV cases were MSM, a population that represented 41% of all newly reported HIV cases among males in Alberta in 2011 [11].

Although, we purposely decided to offer testing at existing outreach sites that were accessed by high risk population represented in the existing outbreak, our study is limited by a nonrandom sample within these settings, which may have resulted in a selection bias and thus may limit the generalizability of our results. Our study participants had accessed Outreach sites and had consented to standard testing which may have inflated the proportion of persons accepting POC testing. However our data does agree with data from other researches at both incarceration sites and MSM sites [29, 30]. Additionally, the majority of those who participated had been tested in the past and the majority of those who tested positive for syphilis were previously treated. This made it difficult to evaluate the impact of introducing syphilis POC testing in these settings; however, we were able to focus on the feasibility of the strategy and the performance of the test in these outreach settings. In summary, our study showed that offering both HIV and syphilis POC testing to at risk populations in outreach settings in Edmonton, Canada was feasible as defined by the high proportion who completed POC testing. Additionally, the SD Bioline 3.0 Syphilis Test and INSTI HIV-1/HIV-2 Antibody test performed well in these settings when compared to standard testing. We found undiagnosed cases of syphilis and HIV and linked them to treatment and care. However, our ability to evaluate the impact and value of syphilis POC testing in these settings was limited by the small sample size, high prevalence of previously treated syphilis, and low prevalence of new cases of infectious syphilis. Future research should evaluate the usefulness of dual syphilis POC tests in similar settings. HIV POC testing may be helpful in reaching undiagnosed HIV infected, particularly when delivered to at risk populations such as MSM in social settings such as bathhouses.

## Figures and Tables

**Table 1:** Characteristics of participants offered syphilis and/or HIV POC testing (*N* = 1,265).

	Accepted (*n* = 1031)	Declined (*n* = 234)	Total (*n* = 1265)	*P* value^a^
Sex				
Female	272 (26.4)	71 (30.3)	343 (27.1)	0.22
Male	759 (73.6)	163 (69.7)	922 (72.9)	

Median age (IQR)	30 (25–39)	29 (24–38)	30 (24–39)	0.52

Testing site				
Corrections	508 (80.0)	127 (20.0)	635	<0.001
Inpatient addictions	291 (85.8)	48 (14.2)	339	
Health facility	39 (73.6)	14 (26.4)	53	
Community-based organization	78 (69.6)	34 (30.4)	112	
MSM	115 (91.3)	11 (8.7)	126	

IQR: interquartile range.

^a^Compares participants who accepted and those who declined using chi square and Mann Whitney tests.

**Table 2:** Demographic and risk behaviour characteristics of individuals who received syphilis and HIV POC testing (*N* = 951).

Characteristic	*n*	%
Male gender	697	73.3
Median age (years, IQR)	30	25–39
Ethnicity		
Aboriginal^a^	406	44.0
Caucasian	449	48.6
Other ethnicities	68	7.4
No permanent address	185	24.2
Sexual partnering		
Heterosexual exclusively	753	79.9
Same sex^b^	189	20.1
Sexual behaviour		
Median age of sexual debut (IQR)	14	13–16
# sexual partners in last 6 months	2	1–3.5
History of sex work (females only)	113	44.5
History of sex work (males only)	6	0.9
Sex with a sex worker (males only)^c^	154	22.1
Substance use		
History of non-IDU	776	81.6
History of IDU	243	25.6
History of sharing drug equipment	556	58.5
Laboratory test results		
Previous HIV test	725	82.6
HIV seropositive	4	0.4
Syphilis seropositive	31	3.3

IQR: interquartile range.

IDU: injection drug use.

^a^Includes First Nations, Inuit, and Métis.

^b^Includes individuals who reported sex with both males and females.

^c^No female participants reported sex with a sex worker.

**Table 3:** Performance characteristics of syphilis and HIV point-of-care testing.

Factor	Positive (%)	Negative (%)	Total (%)	Performance^a^ (%; 95% CI)
Syphilis^b^				
POCT result	29 (2.8)	990^c^ (97.2)	1019 (100.0)	
Standard test Result	34 (3.3)	985 (96.7)	1019 (100.0)	
Sensitivity				85.3 (68.9–95.0)
Specificity				100.0 (99.6–100^d^)
PPV				100.0 (88.1–100^d^)
NPV				99.5 (98.8–99.8)
HIV^e^				
POCT result	6 (0.6)	993^f^ (99.4)	999 (100.0)	
Standard test Result	4 (0.5)	995 (99.6)	999 (100.0)	
Sensitivity				100.0 (39.8–100^d^)
Specificity				99.8 (99.3–100)
PPV				66.7 (22.3–95.7)
NPV				100.0 (99.6–100^d^)

CI: confidence interval; POCT: point-of-care test; PPV: positive predictive value; NPV: negative predictive value.

^a^Standard testing was used as gold standard.

^b^Calculations do not include 1 invalid POCT result (negative on follow-up standard testing).

^c^Does not include 4 negative specimens which did not have standard follow-up testing performed (insufficient quantity (*n* = 2), specimen not labelled (*n* = 1), unable to draw blood (*n* = 1)).

^d^One-sided, 97.5% confidence interval.

^e^Calculations do not include 3 invalid POCT results (negative on follow-up standard testing).

^f^Does not include 9 negative which were indeterminate (*n* = 3) or did not have standard testing performed (insufficient quantity (*n* = 4), or missing specimen (*n* = 2)).
